# Spontaneous retropharyngeal haematoma: a case report

**DOI:** 10.1186/1752-1947-2-8

**Published:** 2008-01-18

**Authors:** Arvind Singh, Enyi Ofo, Vincent Cumberworth

**Affiliations:** 1 Department of Otolaryngology, Northwick Park Hospital, Harrow, UK; 2 Imperial College London, London, UK

## Abstract

**Introduction:**

Spontaneous retropharyngeal haematoma is an unusual condition. It has multiple aetiological factors and can present to a number of specialists including the otolaryngologist.

**Case presentation:**

We describe a case of spontaneous retropharyngeal haematoma which demonstrates the dramatic presentation and emphasises the need for a conservative approach.

**Conclusion:**

It is important to be aware of this unusual condition with its distinct presentation. Surgical intervention should be resisted unless a treatable aetiological factor is found or airway compromise occurs. Most cases will resolve with conservative management.

## Introduction

Retropharyngeal haematoma is a rare entity with multiple aetiological factors. If no cause can be found the condition is labelled as spontaneous retropharyngeal haematoma (SRH) [1]. It has been described too infrequently to determine the prevalence. This is an alarming condition and although associated with life-threatening complications, often the condition resolves without event.

We describe a case of spontaneous retropharyngeal haematoma which demonstrates the dramatic presentation and emphasises the need for a conservative approach.

## Case presentation

**Figure 1:**
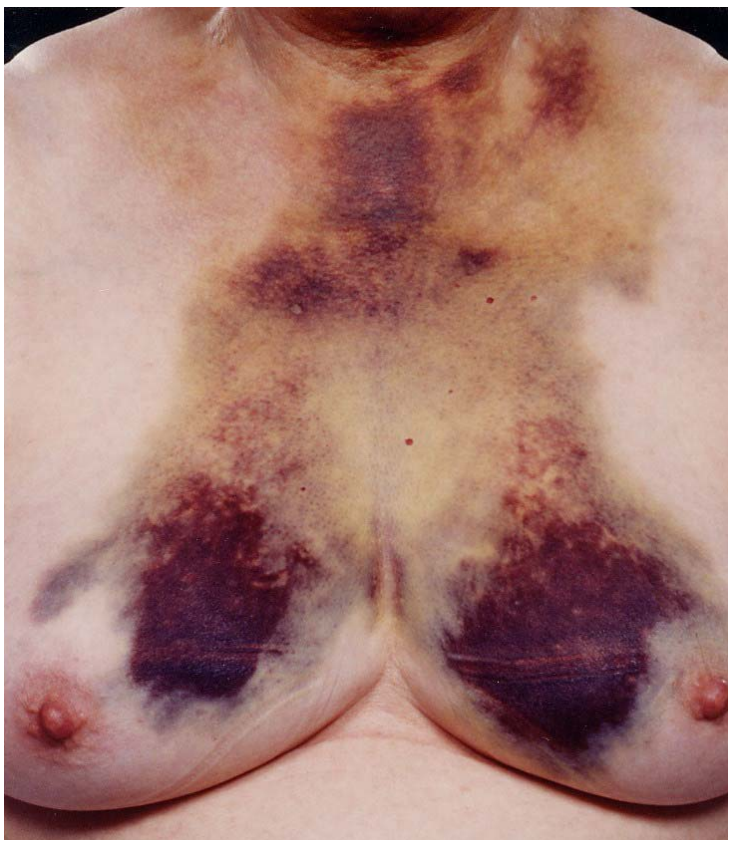
Spontaneous retropharyngeal haematoma: Dramatic bruising seen on the front of the neck and chest wall.

A 61 year old lady presented with a three day history of dysphagia and mild dyspnoea associated with bruising of the neck and front of the chest (Fig. 1). She had no other symptoms and signs. Her only medication was a combination antihypertensive (atenolol/chlortalidone) and her blood pressure was well controlled throughout. She denied any other medication, either self-taken or GP prescribed. A CT scan indicated a mass extending from the oropharynx to below the level of the tracheal bifurcation with some tracheal deviation and narrowing. Haematological tests including a clotting screen were normal as well as a barium swallow. An Aortogram performed five days after the initial presentation was completely normal.

The condition gradually improved and regression of the swelling was apparent on a repeat CT scan ten days later, by which time the external bruising had fully settled. A further CT scan of her chest three months later was completely normal and she had no further problems at all over a four year follow up period.

## Discussion

Retropharyngeal haematoma is associated with a wide variety of aetiologies. These include trauma (central venous cannulation [2], fishbone impaction [3]), haematological issues (anticoagulation [4], Polycythaemia Rubravera [5], hemophilia [6]), neoplasia [7], Epstein-Barr virus [8], vascular aneurysms [9], parathyroid lesions [10]. Spontaneous retropharyngeal haematoma is defined by the absence of any clear aetiology.

The retropharyngeal space is a potential space located immediately posterior to the nasopharynx, oropharynx, hypopharynx, larynx, and trachea. The buccopharyngeal fascia which surrounds the pharynx, trachea, esophagus, and thyroid, forms the anterior border of the retropharyngeal space. Bounded posteriorly by the alar fascia, the retropharyngeal space is limited laterally by the carotid sheaths and parapharyngeal spaces. It extends superiorly to the base of the skull and inferiorly to the mediastinum at the level of the tracheal bifurcation. Infections or blood can track into the mediastinum, neck and anterior chest wall via the interconnecting deep neck spaces.

Clinically SRH can present as a triad of features including superior mediastinal obstruction, anterior tracheal displacement and bruising on the neck within 48 hours subsequently spreading on to the chest wall [11]. Airway obstruction may follow significant superior mediastinal compression and airway intervention in the form of intubation or tracheostomy may be required. The latter can be difficult depending on the extent of bleeding.

The management of SRH is dependent on an understanding of its aetiology and potential complications. Close airway monitoring is essential with the ability for active intervention by intubation or a surgical airway. Surgical evacuation of the haematoma is required in only a minority of cases as spontaneous resolution occurs with in a few weeks. However, there is a reported mortality rate of up to twenty per cent [12].

## Conclusion

Spontaneous retropharyngeal haematoma may present to different disciplines including otorhinolaryngologists and thoracic surgeons. It is important to be aware of this unusual condition with its distinct presentation. Thorough assessment including fibreoptic upper aerodigestive tract visualisation is recommended. Surgical intervention should be resisted unless a treatable aetiological factor is found or airway compromise occurs. Most cases will resolve with conservative management.

## Competing interests

The author(s) declare that they have no competing interests.

## Authors' contributions

Arvind Singh – principal author, researcher, read and approved final manuscript.

Enyi Ofo – co-author and proofreader, read and approved final manuscript.

Vincent Cumberworth – senior author, researcher, proofreader, read and approved final manuscript.

## Consent

Written informed consent was obtained from the patient for publication of this case report and the accompanying image. A copy of the written consent is available for review by the Editor-in-Chief of this journal.
